# Computed tomography based analyses of body mass composition in HER2 positive metastatic breast cancer patients undergoing first line treatment with pertuzumab and trastuzumab

**DOI:** 10.1038/s41598-022-07143-1

**Published:** 2022-03-01

**Authors:** Michela Palleschi, Andrea Prochowski Iamurri, Emanuela Scarpi, Marita Mariotti, Roberta Maltoni, Francesca Mannozzi, Domenico Barone, Giovanni Paganelli, Michela Casi, Emanuela Giampalma, Ugo De Giorgi, Andrea Rocca

**Affiliations:** 1 IRCCS Istituto Romagnolo per lo Studio dei Tumori (IRST) “Dino Amadori”, Meldola, Italy; 2 Radiology Unit, IRCCS Istituto Romagnolo per lo Studio dei Tumori (IRST) “Dino Amadori”, Meldola, Italy; 3 Unit of Biostatistics and Clinical Trials, IRCCS Istituto Romagnolo per lo Studio dei Tumori (IRST) “Dino Amadori”, Meldola, Italy; 4 Nuclear Medicine Unit, IRCCS Istituto Romagnolo per lo Studio dei Tumori (IRST) “Dino Amadori”, Meldola, Italy; 5 Nuclear Medicine Unit, Bufalini Hospital, AUSL Della Romagna, Cesena, Italy; 6 Radiology Department, Bufalini Hospital, AUSL Della Romagna, Cesena, Italy

**Keywords:** Breast cancer, Cancer imaging

## Abstract

Body composition parameters (BCp) have been associated with outcome in different tumor types. However, their prognostic value in patients with HER2-positive metastatic breast cancer (BC) receiving first line treatment with dual anti-HER2 antibody blockade is unknown. Preclinical evidences suggest that adipocytes adjacent to BC cells can influence response to anti-HER2 treatments. We retrospectively analyzed Computed Tomography (CT)-based BCp from 43 patients with HER2-positive metastatic BC who received first line pertuzumab/trastuzumab-based treatment between May 2009 and March 2020. The impact of baseline CT-based BCp on progression-free survival (PFS) was tested using Kaplan–Meier estimates and univariate and multivariate Cox regression models. We found a significantly worse PFS for patients with high baseline subcutaneous fat index (median 7.9 vs 16.1 months, p = 0.047, HR = 2.04, 95%CI 1–4.17) and for those with high total abdominal fat index (8.1 vs 18.8 months, p = 0.030, HR = 2.17, 95%CI 1.06–4.46). Patients with baseline sarcopenia did not show shorter PFS compared to those without sarcopenia (10.4 vs 9.2 months, p = 0.960, HR = 0.98, 95%CI 0.47–2.03). Total abdominal fat index remained a significant predictor of PFS at multivariate analysis. Our findings suggest that a high quantity of total abdominal fat tissue is a poor prognostic factor in patients receiving trastuzumab/pertuzumab-based first-line treatment for HER2-positive metastatic BC.

## Introduction

The association between obesity and early breast cancer (BC) has been described in various clinical trials: in both pre- and postmenopausal patients the excess of body weight is correlated with higher relapse and poorer survival with respect to normal-weight individuals.

A number of meta-analyses have confirmed the negative prognostic impact of obesity, which is more evident in patients with estrogen receptor (ER)-positive BC^1–3^.

The relation between body mass index (BMI) and prognosis has yet to be clarified for metastatic disease, although some studies have hypothesized possible protective effect of excess body weight, in conflict with data reported in early-stage BC^4–6^. This phenomenon called ‘’obesity paradox’’ has also been described in other tumor types, in particular gastrointestinal malignancies, and could be explained by the fact that body composition is highly variable between individuals sharing same BMI.

Current evidence suggests that a more in-depth analysis of body composition might disprove this obesity protective effect^7^: the better prognosis seen in patients with higher BMI may be attributable to differences in body composition parameters including fat tissue distribution and muscle mass. Further research is warranted to better understand the complex relationship between BMI and treatment outcomes in patients with metastatic BC.

Sarcopenia, or the age-related loss of skeletal muscle mass, is acknowledged as an important independent indicator of poor prognosis in cancer patients. Sarcopenia in BC is known to be a predictor of poor survival and is correlated with a higher incidence of treatment-derived toxicity and a shorter time to progression. If we focus on specific BC subtypes, there is a lack of the literature data regarding BCp and outcomes.

Contradictory results have been reported on the effect of excess body weight on outcome of patients treated for metastatic HER2-positive BC. Parolin et al. and Krasniqi et al.^8,9^ found that higher BMI was associated with poor prognosis in patients receiving trastuzumab-based regimens and TDM-1. Conversely, in a recent multicenter retrospective cohort study of BC patients undergoing first-line trastuzumab-based treatment BMI was not correlated with progression-free survival (PFS) (adjusted hazard ratio [HR] = 0.88, 95% CI 0.66–1.17, p = 0.387) or overall survival (OS) (adjusted HR = 0.88, 95% CI 0.59–1.31, p = 0.525)^10^.

As previously reported in several studies, BMI may not be the best for the assessment of body fat^7^. Although dual X-ray absorptiometry (DEXA) is considered the reference standard for evaluating body composition, it is seldom used in clinical practice.

It is known that Computed Tomography (CT)-based regional analysis of muscle and fat tissue in the area of the third lumbar vertebra is tied to whole-body fat and muscle mass, making it a convenient method to study body composition in cancer patients given that CTs are routinely carried out to monitor response to treatment.

We suspect that anti-HER2 drugs may be less active in obese patients, reducing their response to these agents due to a modification in their body composition parameters (decrease in lean mass and increase in fat mass).

The present study examined the correlation of baseline BCp on response to first-line pertuzumab- and trastuzumab-based treatment in patients with HER2-positive metastatic BC^11,12^.

## Results

### Patients cohort

The final cohort included 43 patients (median age 58 years, range 52–64 years), with histological diagnosis of invasive ductal carcinoma in 36 cases (85.7%), invasive lobular carcinoma in 2 cases (4.8%, both were pleomorphic lobular neoplasms) and other histological type (i.e. non-ductal, non-lobular) in 5 cases (9.5%).

At the time of diagnosis, BC stage was I to III in 25 cases (58.1%) and IV in 18 cases (41.9%). ER at diagnosis were positive in 28 cases (65.1%) and PgR in 13 cases (30.2%). Out of 43 patients, 33 (76.7%) were in menopause. All patients received taxane based chemotherapy (paclitaxel, twelve administrations at a dose of 80 mg/m^2^ every 7 days, or docetaxel six cycles at a dose of 75 mg/m^2^ every 21 days) and trastuzumab plus pertuzumab for six cycles.

All hormone-receptor positive patients received as maintenance endocrine treatment (plus dual anti-HER2 antibody blockade) in according to their menopausal status; in particular, ten of these underwent to exemestane plus luteinizing hormone-releasing hormone (LHRH) analogs and 33 letrozole, based on physician’s choice. At the beginning of the therapy, the ECOG performance status was 0 in 39 patients (90.7%) and from 1 to 2 in 4 patients (9.3%). Twenty-one patients (48.8%) had normal BMI, 16 were overweight (25 ≤ BMI < 30, 37.2%) and 6 were obese (BMI ≥ 30, 14%). Fifteen patients (34.9%) demonstrated baseline sarcopenia whereas 28 patients (65.1%) had a normal skeletal muscle index. The agreement between the two readers on the BCp measurements was excellent (0.998 95% CI 0.997–0.998, where 0 is no agreement and 1 is perfect agreement).

All additional patient characteristics and body composition parameters are described in Table 1.

**Table 1:** Patients’ clinical and metabolic parameters at the diagnosis and at the beginning of the therapy.

	N (%)
**Parameters at the diagnosis**	
Age median (inter quartile range)	54 (47–61)
Menopausal status	Pre 10 (23.3)
	Post 33 (76.7)
Histology	Ductal 36 (83.7)
	Lobular 2 (4.7)
	Other 5 (11.6)
Stage	I-III 25 (58.1)
	IV 18 (41.9)
Surgery	No 11 (26.8)
	Yes 30 (73.2)
Er (%)	Negative (0) 15 (34.9)
	Positive (≥ 1) 28 (65.1)
PgR(%)	Low (≤ 20) 30 (69.8)
	High (> 20) 13 (30.2)
Ki67 (%)	Low (≤ 20) 9 (21.9)
	High (> 20) 32 (78.1)
	Missing 2
**Parameters at the beginning of the therapy**	
Age	58 (52–64)
Weight median (inter quartile range)	65 (59–74)
ECOG performance status	0 39 (90.7)
	1–2 4 (9.3)
BMI	< 25 21 (48.8)
	25–30 16 (37.2)
	> 30 6 (14.0)
**Body composition parameters**	**Median (inter quartile range)**
SM (skeletal muscle; cm^2^)	110.18 (97.43–124.58)
SF (subcutaneous fat; cm^2^)	225.06 (150.93–285.02)
VF (visceral fat; cm^2^)	93.77 (44.47–140.79)
TAFT (Total abdominal fat; cm^2^)	299.11 (222.28–399.89)
SMI (skeletal muscle index; cm^2^/m^2^)	42.26 (36.02–45.04)
SFI (subcutaneous fat index; cm^2^/m^2^)	82.97 (55.68–118.63)
VFI (visceral fat index; cm^2^/m^2^)	37.10 (18.04–60.14)
TAFTI (Total abdominal fat index; cm^2^/m^2^)	118.82 (86.54–157.39)

### Univariate analysis

**Figure 1:**
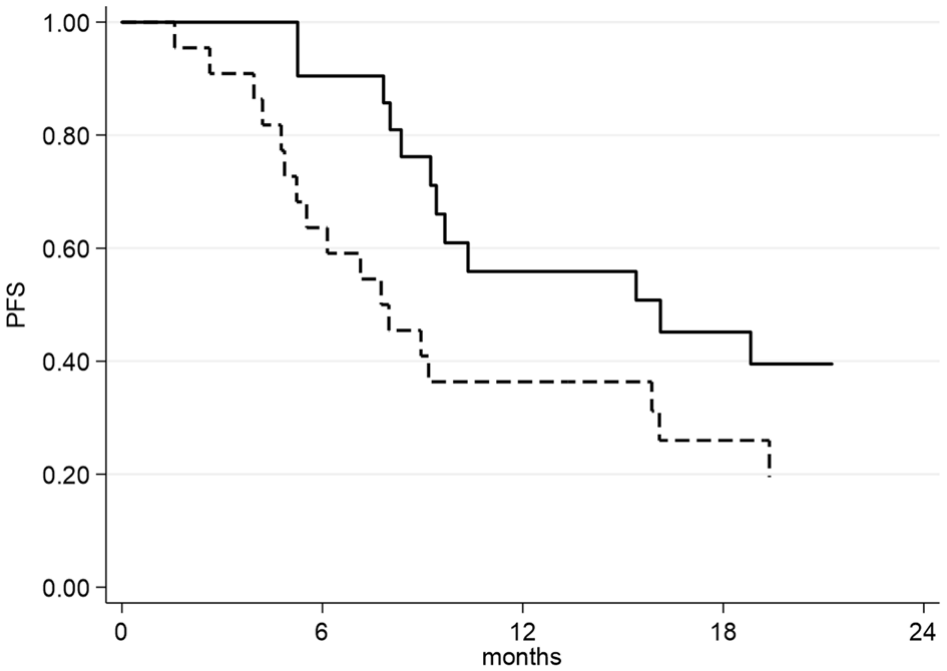
Progression-free Survival as a function of Subcutaneous Fat Index (SFI). Continuous line represents patients with normal (below the median value: 82.97 cm^2^/m^2^) SFI, dashed line represents patients with high (equal or above the median value: 82.97 cm^2^/m^2^) SFI.

**Figure 2:**
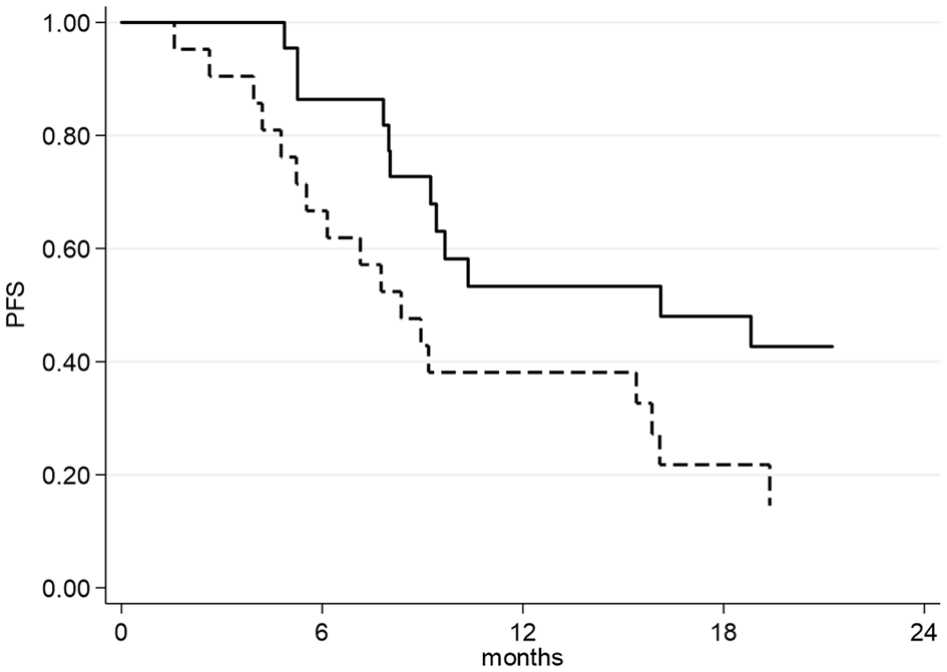
Progression-free Survival as a function of Total Abdominal Fat Index (TAFTI). Continuous line represents patients with normal (below the median value: 118.82 cm^2^/m^2^) TAFTI, dashed line represents patients with high (equal or above the median value: 118.82 cm^2^/m^2^) TAFTI.

The median PFS was 9.7 months (95% CI 8.0–16.1) with a median follow-up of 33 months. The median time between the baseline CT and the beginning of therapy was 14 days. Univariate analysis demonstrated a significantly worse PFS for patients with high baseline subcutaneous fat index (7.9 vs 16.1 months, p = 0.047, HR = 2.04, 95% CI 1–4.17, Fig. 1) and high total abdominal fat index (8.1 vs 18.8 months, p = 0.030, HR = 2.17, 95% CI 1.06–4.46, Fig. 2).

Conversely, patients with baseline sarcopenia did not show shorter PFS compared to patients with regular SMI (10.4 vs 9.2 months, p = 0.960, HR = 0.98, 95% CI 0.47–2.03). Likewise, overweight/obese patients (BMI ≥ 25) did not demonstrate a reduced PFS compared to normal body weight patients (9.2 vs 9.7 months, p = 0.815, HR = 0.92, 95% CI 0.45–1.86).

Among the clinical parameters, expression of ER positively affects PFS (9.7 vs 6.2 months, p = 0.035, HR = 0.31, 95% CI 0.10–0.98). All the other clinical and metabolic parameters considered, such as age, Ki67, LDL, menopausal status and sites of metastasis, did not show any significant correlation with PFS. Result of the univariate analysis are summarized in Table 2.

**Table 2:** Univariate analysis results for all the parameters considered in the study. Significant values are in bold.

	Median PFS months (95% CI)	logrank p value	HR (95% CI)
Overall	9.7 (8.0–16.1)	–	–
**Age (years)**			
< 58	13.1 (5.3–18.8)		Reference
≥ 58	9.2 (7.8–55.5)	0.891	0.95 (0.47–1.93)
**Stage**			
I-III	15.4 (7.8–18.8)		Reference
IV	9.2 (5.5–25.0)	0.886	1.06 (0.49–2.29)
**Surgery**			
No	8.4 (3.9–25.0)		Reference
Yes	15.4 (8.0–19.4)	0.303	0.66 (0.30–1.46)
**Histology**			
Ductal	9.2 (7.8–16.1)		Reference
Other	55.5 (3.9–nr)	0.045	0.25 (0.06–1.07)
**Metastatic site**			
Other	9.2 (6.2–18.8)		Reference
Only Bone	16.1 (8.0–55.5)	0.505	0.75 (0.32–1.75)
**LDL (mg/dL)**			
Normal (< 100)	9.4 (5.3–25.0)		Reference
High (> 100)	9.7 (6.2–55.5)	0.854	0.93 (0.41–2.09)
**Triglycerides (mg/dL)**			
Normal (< 150)	9.4 (5.3–19.4)		Reference
High (≥ 150)	9.7 (7.8–nr)	0.223	0.61 (0.27–1.37)
**Menopausal status**			
Pre	8.7 (2.6–16.1)		Reference
Post	15.4 (8.0–25.0)	0.139	0.55 (0.25–1.22)
**ER (%)**			
Negative (0)	6.2 (4.2–9.4)		Reference
Positive (≥ 1)	9.7 (5.3–nr)	**0.035**	0.31 (0.10–0.98)
**PgR (%)**			
Low (≤ 20)	8.1 (4.9–9.7)		Reference
High (> 20)	nr	0.073	0.33 (0.09–1.18)
**Ki67 (%)**			
Low (≤ 20)	12.5 (3.9–nr)		Reference
High (> 20)	7.8 (4.2–18.8)	0.421	1.60 (0.50–5.10)
**BMI (kg/m**^**2**^**)**			
< 25	9.7 (5.3–19.4)		Reference
≥ 25	9.2 (7.1–16.1)	0.815	0.92 (0.45–1.86)
**SMI (cm**^**2**^**/m**^**2**^**)**			
Normal (> 40)	9.2 (6.2–25.0)		Reference
Sarcopenia (< 40)	10.4 (7.8–18.8)	0.960	0.98 (0.47–2.03)
**SFI (cm**^**2**^**/m**^**2**^**)**			
Normal (< 82.97)	16.1 (9.2–nr)		Reference
High (> 82.97)	7.9 (4.9–16.1)	**0.047**	2.04 (1.00–4.17)
**VFI (cm**^**2**^**/m**^**2**^**)**			
Normal (< 37.1)	10.4 (8.0–18.8)		Reference
High (> 37.1)	9.1 (6.2–19.4)	0.939	1.03 (0.50–2.11)
**TAFTI (cm**^**2**^**/m**^**2**^**)**			
Normal (< 118.82)	18.8 (9.2–nr)		Reference
High (> 118.82)	8.1 (4.9–15.9)	**0.030**	2.17 (1.06–4.46)

*PFS* progression-free survival, *HR* hazard ratio (from Cox regression models), *CI* confidence interval, *ER* estrogen receptor, *PgR* progestin receptor, *BMI* body mass index, *SMI* skeletal muscle index, *SFI* subcutaneous fat index, *VFI* visceral fat index, *TAFTI* Total abdominal fat index, *nr* not reached.

High subcutaneous fat index (p = 0.013, HR = 4.57, 95% CI 1.38–15.08) and high total abdominal fat index (p = 0.002, HR 8.56, 95% CI 2.19–33.47) demonstrated an even higher association with reduction of PFS in the subgroup of patients with the novo metastatic disease (Table 3, stage IV), whereas no significant effect was found in patients with stage I–III at diagnosis. Interestingly, Visceral Fat Index showed a significant qualitative interaction with stage (p = 0.024), with an opposite effect on PFS, in patients with stage I–III compared to those with de novo stage IV disease, that deserves further investigation. While the interaction between subcutaneous fat index and stage was not significant (p = 0.1), high total abdominal fat index showed a significant quantitative interaction with stage (p = 0.026).

**Table 3:** Correlation between PFS and body composition parameters classifying patients based on the stage at the diagnosis. Significant values are in bold.

Parameter	Value	Stage I–III		Stage IV	
		HR (95% CI)	p-value	HR (95% CI)	p-value
BMI (kg/m^2^)	< 25	Reference		Reference	
	≥ 25	0.56 (0.22–1.43)	0.223	1.73 (0.56–5.31)	0.337
SFI (cm^2^/m^2^)	Normal (< 82.97)	Reference		Reference	
	High (> 82.97)	1.23 (0.48–3.10)	0.666	4.57 (1.38–15.08)	**0.013**
SMI (cm^2^/m^2^)	Normal (> 40)	Reference		Reference	
	Sarcopenia (< 40)	1.30 (0.51–3.28)	0.581	0.64 (0.17–2.39)	0.512
VFI (cm^2^/m^2^)	Normal (< 37.1)	Reference		Reference	
	High (> 37.1)	0.53 (0.20–1.39)	0.195	2.80 (0.90–8.72)	0.075
TAFTI (cm^2^/m^2^)	Normal (< 118.82)	Reference		Reference	
	High (> 118.82)	1.14 (0.45–2.90)	0.775	8.56 (2.19–33.47)	**0.002**

*HR* hazard ratio (from Cox regression models), *CI* confidence interval, *BMI* body mass index, *SMI* skeletal muscle index, *SFI* subcutaneous fat index, *VFI* visceral fat index, *TAFTI* Total abdominal fat index.

Moreover, univariate and multivariate analysis for Overall Survival (OS) were performed but no significant correlations between all variables considered in this study and the OS were found (tables included in the supplementary materials).

### Multivariate and multiparametric analysis

At multivariate analysis, that included ER, SFI and TAFTI as significant univariate predictors and menopausal status and BMI as clinically relevant variable, only ER, TAFTI and menopausal status showed an independent significant association with PFS: high total abdominal fat index as a detrimental factor (p = 0.05, HR = 3.47, 95% CI 1.00–13.04), ER-positivity and post-menopausal status as protective factors (p = 0.017, HR = 0.33, 95% CI 0.14–0.82, and p = 0.021, HR = 0.36, 95% CI 0.15–0.86, respectively) (Table 4). Additionally, multivariate analysis was performed for the OS, but no significant correlation were found (table included in the supplementary materials).

**Table 4:** Cox multivariate analysis of progression-free survival (PFS).

	HR (95% CI)	p-value
**TAFTI (cm**^**2**^**/m**^**2**^**)**		
Normal (< 118.82)	Reference	
High (> 118.82)	3.47 (1.00–13.04)	0.050
**SFI (cm**^**2**^**/m**^**2**^**)**		
Normal (< 82.97)	Reference	
High (> 82.97)	1.51 (0.49–4.63)	0.470
**BMI (kg/m**^**2**^**)**		
< 25	Reference	
≥ 25	0.70 (0.26–1.90)	0.481
**ER (%)**		
Negative (0)	Reference	
Positive (≥ 1)	0.33 (0.14–0.82)	0.017
**Menopausal status**		
Pre	Reference	
Post	0.36 (0.15–0.86)	0.021

*TAFTI* Total abdominal fat index, *SFI* Subcutaneous fat Index, *BMI* Body mass index, *ER* Estrogen receptor.

Multiparametric analysis demonstrated a strong correlation between BMI and TAFTI, SFI and VFI (p < 0.0001), as well as with SMI (p = 0.005). Additionally, a strong correlation was found between ER and PgR (p < 0.0001) and between weight and most of the body composition parameters (Table 5).

**Table 5:** Spearman correlation analysis of the body composition parameters.

	SFI	VFI	TAFTI	BMI	ER	PgR	Ki67	Weight
	r_s_ p	r_s_ p	r_s_ p	r_s_ p	r_s_ p	r_s_ p	r_s_ p	r_s_ p
SMI	0.39 0.009	0.37 0.016	0.42 0.005	0.42 0.005	− 0.04 0.810	− 0.13 0.400	− 0.01 0.958	0.35 0.024
SFI	–	0.63 < 0.0001	0.94 < 0.0001	0.78 < 0.001	0.23 0.138	0.16 0.306	− 0.03 0.862	0.54 0.0002
VFI	–	–	0.84 < 0.0001	0.75 < 0.0001	0.23 0.146	0.12 0.455	− 0.18 0.273	0.56 0.0001
TAFTI	–	–	–	0.85 < 0.0001	0.27 0.082	0.16 0.302	− 0.08 0.628	0.58 < 0.0001
BMI	–	–	–	–	0.31 0.044	0.27 0.083	− 0.09 0.563	0.83 < 0.0001
ER	–	–	–	–	–	0.69 < 0.0001	− 0.43 0.005	0.39 0.012
PgR	–	–	–	–	–	–	− 0.17 0.280	0.40 0.009
Ki67	–	–	–	–	–	–	–	− 0.16 0.338

*BMI* body mass index, *SMI* skeletal muscle index, *SFI* subcutaneous fat index, *VFI* visceral fat index, *TAFTI* Total abdominal fat index, *ER* estrogen receptor, *PgR* progestin receptor.

## Discussion

Breast cancer is a heterogeneous disease, whose development entails the accumulation of various genetic and epigenetic alterations, ultimately leading to deregulated intracellular signaling. However, is currently evident that factors such as excess body weight have an important promoting role^13,14^.

In the early breast cancer setting, a significant amount of data indicates that overweight (BMI 25–29.9 kg/m^2^) or obesity (BMI ≥ 30 kg/m^2^) are associated with shorter survival^5,15^. On the contrary, in metastatic BC patients, the available evidence is scant and discordant^16–20^. The evidence is even more inconsistent when the various molecular subtypes are considered. To the best of our knowledge, only three studies have been published to date on HER2‐positive metastatic BC (HER2 + mBC), all with contrasting results^8–10^, and only one^9^ included findings on the newer anti‐HER2 drugs pertuzumab and trastuzumab emtansine (T‐DM1). In our series, BMI was not associated with outcome, in line with the findings of Martin and colleagues.

However, numerous preclinical studies have suggested that adipose tissue may influence response to anti-HER2 treatment. Adipocytes produce several paracrine and endocrine factors known as adipocytokines, including leptin, adiponectin, tumor necrosis factor (TNF)-α and interleukin 6, potentially affecting tumor growth. Leptin receptor and HER2 are frequently co‐expressed in breast cancer cell lines and tumors, interacting with each other and leading to HER2 phosphorylation in response to leptin exposure^21^. Leptin can also transactivate HER2 via epidermal growth factor receptor (EGFR) and Janus-activated kinase 2 activation^22^. Another study^23^ hypothesized that leptin sustained HER2 protein levels via an upregulation of the heat shock protein 90 chaperone expression.

Duong et al.^24^ observed that pre-adipocytes and adipocytes suppressed trastuzumab-mediated antibody-dependent cellular cytotoxicity in breast cancer cells through soluble factor secretion, leading to a protective effect on tumor cells.

Several preclinical studies have evaluated mechanisms of resistance to trastuzumab. One such mechanism is the activation of signaling pathways, in particular the insulin-like growth factor 1 (IGF-1) receptor, which is also known to impede trastuzumab-mediated growth inhibition in breast cancer cells. Saxena et al.^25^ described a bidirectional crosstalk between leptin and IGF‐1 signaling mediated by transactivation of EGFR promoting BC cell migration and invasion.

Two large meta-analyses of 43 and 82 international clinical studies reported a poorer outcome for very overweight early BC patients with respect to non-obese patients regardless of menopausal or hormone receptor status. The evidence is more controversial in HER2-positive BC. We conducted a retrospective study on 53 HER2-positive early BC patients, 23 of whom had locoregional or distant relapse, but found no impact of BMI and ER status on the recurrence risk. Conversely, higher percentage of relapse in patients with BMI ≥ 25 kg/m^2^ and ER-negative status was reported by Cantini et al.^26,27^. The impact of BMI may differ according to the chemotherapy regimen, as shown by a recent retrospective analysis of a Breast International Group adjuvant trial^28^ demonstrating a differential response to docetaxel, but not to other chemotherapy regimens, based on BMI.

As mentioned above, BMI alone may not be the most suitable method to assess body composition. Recently, interest has arisen in the role of low muscle mass in cancer patients^29,30^. Individuals with low muscle mass appear to have poorer survival than those with normal muscle mass, especially in gastrointestinal cancer^31–33^.

Muscle mass is generally determined by CT scans, which are considered the gold standard for measuring muscle parameters. It is known that the muscle cross-sectional area is strongly correlated with total body muscle mass, and muscle measurement can easily be performed using CT images acquired during routine follow-up. Although the prognostic impact of skeletal muscle measures has been studied in different tumor types, this is relatively unexplored area in BC. The research field of body composition is especially clinically relevant because muscle parameters might help to estimate prognosis and possibly to predict responsiveness to treatments.

In Caan et al.’s study, the largest to date on patients with early BC, muscle and fat mass, evaluated clinically by CT scan, were found to be more strongly correlated with survival than BMI. The authors point out that sarcopenia and adiposity are both important risk factors and should be appraised together when assessing risk of relapse. The HEAL study also reported that sarcopenia, evaluated by DEXA, was linked to overall mortality in 471 patients with stage I-IIIA BC^34^. An impact of muscle mass on chemotherapy toxicity has been reported in a prospective trial on early BC patients treated with taxanes and anthracyclines regimens^35^.

The majority of data on metastatic disease come from smaller studies focusing on the impact of sarcopenia in patients undergoing chemotherapy^36^. In a study of 55 patients treated with capecitabine for metastatic BC^37^, those who had low muscle mass (calculated using total abdominal muscle area measurement) had a threefold higher risk of toxicity, e.g. diarrhea and stomatitis, and a shorter time to progression. Rier et al. reported that sarcopenia does not portend a poorer prognosis in patients treated with anthracycline and taxane-based chemotherapy regimens. Our findings confirm same results: sarcopenia was not associated with shorter PFS^38^.

To our knowledge, this is the first study that analyzes the association between body composition parameters and response to dual anti-HER2 antibody blockade regimens for metastatic HER2-positive breast cancer. We observed a shorter PFS in patients with increased amounts of total abdominal fat tissue, while muscle mass did not affect outcome.

Our findings differ from those highlighted by Franzoi et al. in patients receiving endocrine therapy plus cyclin-dependent kinase (CDK) 4/6 inhibitors for hormone receptor-positive, HER2-negative metastastic BC: their results showed an association between sarcopenia and a worse PFS, while ours didn’t. In our series, total visceral fat is the most important factor related to worse outcome, and it might suggest that in HER2-positive breast cancer disease total abdominal fat tissue could have a key role to predict response to newly anti-HER2 treatments, as already shown in preclinical models^39^.

Dual anti-HER2 antibody blockade with trastuzumab and pertuzumab in association with a taxane is now recommended as first-line systemic treatment in patients with HER2-positive metastatic BC following findings a survival benefit in the Cleopatra Study^11^. A recent update of this trial revealed an association between progesterone receptor expression and outcome. We found a similar trend for progestin receptor and a clearer impact of ER on outcome. The impact of BMI or body composition parameters was not analyzed in the Cleopatra trial^12^.

Our findings provide additional information regarding total abdominal visceral fat tissue as a potential poor prognostic factor in HER2-positive metastatic BC, within the context of dual anti-HER2 antibody blockade treatment. Given that body fat and muscle mass represent modifiable elements, programs that aim to promote or improve exercise and nutritional assessment before therapy should be offered to BC patients.

Our study is exploratory, with limitations due to its retrospective nature and limited sample size. Furthermore, we were unable to recover data on diet, malnutrition, physical activity, sedentary habits and comorbidities, which can influence body composition parameters and their changes over time. Further studies are warranted to better understand the impact of body composition parameters, and their changes over time, on treatment outcomes in the different BC subtypes.

## Materials and methods

### Patient selection

Our retrospective study was performed in accordance with the ethical standards laid down in the 1964 Declaration of Helsinki and was approved by the Medical Scientific Committee of Istituto Scientifico Romagnolo per lo Studio e la Cura dei Tumori (IRST) IRCCS and the Ethical Committee of Area Vasta Romagna (C.E.ROM: Comitato Etico della ROMagna, Italy; approval number L3P1574). Informed consent was waived by the Ethical Committee of Area Vasta Romagna (C.E.ROM: Comitato Etico della ROMagna, Italy). From our hospital database we retrieved data (March 3rd, 2020) on all patients treated with first-line pertuzumab and trastuzumab for metastatic HER2-positive BC between May 2009 and July 2020. The methods for HER2 testing used in this study were rigorously adherent to the ASCO/CAP guidelines for 2007, 2013, 2015 and 2018, since the time window of this study is 11 years.

**Figure 3:**
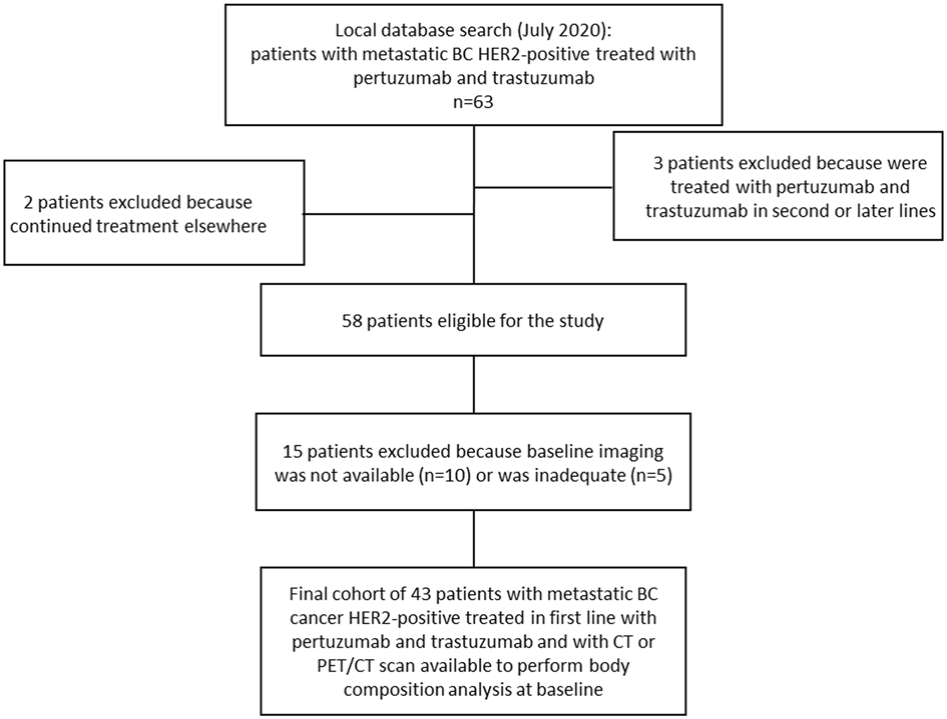
Flow chart of patient selection.

Of 63 patients found, 3 received trastuzumab plus pertuzumab in second or later lines, and 2 continued treatment elsewhere, and were excluded from the analysis. Other 15 patients were excluded because baseline imaging was not available (n = 10) or inadequate (n = 5). The final cohort was made up of 43 patients (Fig. 3).

### Body composition parameters assessment

All patients underwent a baseline CT or positron emission tomography (PET)-CT scan as close as possible to the beginning of treatment with anti‐HER2 agents (not more than 9 weeks before and 1 week after the start of treatment).

To assess body mass composition, a single CT slice at the level of the third lumbar vertebral body, possibly with both transverse processes depicted, was processed using a specific DICOM-viewer software (OsiriX© v.11.0.0; Pixmeo, Geneve, Switzerland). To standardize the measurements of the BCp, all the CT slice analyzed had these characteristics: acquired at 100–120 kV with variable mA, soft tissue reconstruction algorithm, matrix of 512 × 512 and reconstructed slice thickness of 5 mm. On this slice, using a built-in application enabling a semi-automated tissue demarcation based on density thresholds, the total cross-sectional area of fat tissues (TAFT) was calculated by summing the areas of visceral fat tissue (VF), considering a density threshold from − 150 to − 50 Hounsfield Units and excluding visceral organs, and subcutaneous and intramuscular fat (SF), using a density threshold from − 190 to − 30 Hounsfield Units. Similarly, in the same slice, considering a density threshold from − 29 to + 150 Hounsfield Units, the total cross-sectional area of skeletal muscle (TMA) was evaluated (including paraspinal, psoas, and abdominal wall musculature and excluding bones).

All the measurements were performed by a single reader (API), who manually corrected tissue segmentations when necessary, unaware of the final PFS of patients, to avoid any bias.

To confirm these measurements and verify inter-observer variability, another reader (DB), blinded to the BCp obtained by the first reader, independently performed all the measurements and the agreement between the two readers was assessed.

Patient heights were used to calculate the Skeletal Muscle Index (SMI) as follows^40^:

$${\varvec{S}}{\varvec{M}}{\varvec{I}} \frac{{cm}^{2}}{{m}^{2}}=\frac{TMA ({cm}^{2})}{{height }^{2}\, ({m}^{2})}$$

Similarly, sub-cutaneous fat, visceral fat and total abdominal fat were corrected for patient’s height and indexes were obtained (SFI, VFI, and TAFTI).

### Metabolic and clinical parameters

From patient’s medical records, we retrieved several metabolic and clinical baseline parameters such as age, height, weight, BMI, low density lipoprotein (LDL) cholesterol and triglyceride serum levels, menopausal status, ECOG performance status. Pathology data, including histological type, disease stage, Ki67, ER and progestin receptor (PgR) expression levels, were also obtained.

### Definition of parameters' categories

While sarcopenia for BC patients is well defined in the literature (SMI < 40)^41^, the remaining body composition parameters (SFI, VFI, and TAFTI) were categorized calculating the median values of the whole cohort and classified in two categories: low (below median value) and high (equal or above the median value). Metabolic and Clinical Parameters were dichotomized using well established thresholds values^42^.

### Statistical analysis

Progression-free Survival (PFS) was defined as the time elapsed between the beginning of treatment with anti‐HER2 agents and radiologically confirmed disease progression according to RECIST v1.1 or death from any causes, and was tested using Kaplan–Meier estimates. Survival curves were compared using longrank test for univariate analysis, while Cox proportional-hazard regression was used for multivariate analysis. p values < 0.05 were considered statistically significant. Intraclass correlation coefficient test was used to assess inter-observer agreement for the measurements of the body composition parameters.

All analyses were performed using SAS (version 9.4 SAS Institute, Cary, Nc, USA).

## Supplementary Information

Supplementary Information 1.

Supplementary Information 2.

## Data Availability

The authors declare that data supporting the findings of this study are included in this published article and its supplementary information files. Patient’s medical records and DICOM images cannot be shared due to the highly sensitive nature of this data.

## References

[1] Yerushalmi R (2017). Impact of baseline BMI and weight change in CCTG adjuvant breast cancer trials. Ann. Oncol..

[2] Cho WK (2018). Effect of body mass index on survival in breast cancer patients according to subtype, metabolic syndrome, and treatment. Clin. Breast Cancer.

[3] Chan DSM (2014). Body mass index and survival in women with breast cancer-systematic literature review and meta-analysis of 82 follow-up studies. Ann. Oncol..

[4] Ewertz M (2012). Obesity and risk of recurrence or death after adjuvant endocrine therapy with letrozole or tamoxifen in the breast international group 1–98 trial. J. Clin. Oncol..

[5] Kroenke CH, Chen WY, Rosner B, Holmes MD (2005). Weight, weight gain, and survival after breast cancer diagnosis. J. Clin. Oncol..

[6] Kamineni A (2013). Body mass index, tumor characteristics, and prognosis following diagnosis of early-stage breast cancer in a mammographically screened population. Cancer Causes Control.

[7] Trestini I (2018). Clinical implication of changes in body composition and weight in patients with early-stage and metastatic breast cancer. Crit. Rev. Oncol. Hematol..

[8] Parolin V (2010). Impact of BMI on clinical outcome of HER2-positive breast cancer. J. Clin. Oncol..

[9] Krasniqi E (2020). Impact of BMI on HER2+ metastatic breast cancer patients treated with pertuzumab and/or trastuzumab emtansine. Real-world evidence. J. Cell Physiol..

[10] Martel S (2018). Impact of body mass index on the clinical outcomes of patients with HER2-positive metastatic breast cancer. Breast.

[11] Swain SM (2015). Pertuzumab, trastuzumab, and docetaxel in HER2-positive metastatic breast cancer. N. Engl. J. Med..

[12] Swain SM (2020). Pertuzumab, trastuzumab, and docetaxel for HER2-positive metastatic breast cancer (CLEOPATRA): End-of-study results from a double-blind, randomised, placebo-controlled, phase 3 study. Lancet Oncol..

[13] Calle EE, Kaaks R (2004). Overweight, obesity and cancer: Epidemiological evidence and proposed mechanisms. Nat. Rev. Cancer.

[14] Calle EE, Rodriguez C, Walker-Thurmond K, Thun MJ (2003). Overweight, obesity, and mortality from cancer in a prospectively studied cohort of US adults. N. Engl. J. Med..

[15] Cleveland RJ (2007). Weight gain prior to diagnosis and survival from breast cancer. Cancer Epidemiol. Biomarkers Prev..

[16] Gennari A (2013). Body mass index and prognosis of metastatic breast cancer patients receiving first-line chemotherapy. Cancer Epidemiol. Biomarkers Prev..

[17] Jung SY (2012). Factors associated with mortality after breast cancer metastasis. Cancer Causes Control.

[18] Pizzuti L (2018). Body mass index in HER2-negative metastatic breast cancer treated with first-line paclitaxel and bevacizumab. Cancer Biol. Ther..

[19] von Drygalski A (2011). Obesity is an independent predictor of poor survival in metastatic breast cancer: Retrospective analysis of a patient cohort whose treatment included high-dose chemotherapy and autologous stem cell support. Int. J. Breast Cancer.

[20] Zielinski C (2016). Bevacizumab plus paclitaxel versus bevacizumab plus capecitabine as first-line treatment for HER2-negative metastatic breast cancer (TURANDOT): primary endpoint results of a randomised, open-label, non-inferiority, phase 3 trial. Lancet Oncol..

[21] Fiorio E (2008). Leptin/HER2 crosstalk in breast cancer: In vitro study and preliminary in vivo analysis. BMC Cancer.

[22] Soma D (2008). Leptin augments proliferation of breast cancer cells via transactivation of HER2. J. Surg. Res..

[23] Giordano C (2013). Leptin increases HER2 protein levels through a STAT3-mediated up-regulation of Hsp90 in breast cancer cells. Mol. Oncol..

[24] Duong MN (2015). Adipose cells promote resistance of breast cancer cells to trastuzumab-mediated antibody-dependent cellular cytotoxicity. Breast Cancer Res..

[25] Saxena NK (2008). Bidirectional crosstalk between leptin and insulin-like growth factor-I signaling promotes invasion and migration of breast cancer cells via transactivation of epidermal growth factor receptor. Cancer Res..

[26] Maltoni R (2020). Are BMI and negative hormone receptors prognostic factors in HER2(+) early-stage breast cancer?. Clin. Breast Cancer.

[27] Cantini L (2020). Body mass index and hormone receptor status influence recurrence risk in HER2-positive early breast cancer patients. Clin. Breast Cancer.

[28] Desmedt C (2020). Differential benefit of adjuvant docetaxel-based chemotherapy in patients with early breast cancer according to baseline body mass index. J. Clin. Oncol..

[29] Yip C (2015). Imaging body composition in cancer patients: visceral obesity, sarcopenia and sarcopenic obesity may impact on clinical outcome. Insights Imaging.

[30] Kazemi-Bajestani SM, Mazurak VC, Baracos V (2016). Computed tomography-defined muscle and fat wasting are associated with cancer clinical outcomes. Semin. Cell Dev. Biol..

[31] Huang DD (2015). Sarcopenia, as defined by low muscle mass, strength and physical performance, predicts complications after surgery for colorectal cancer. Colorectal Dis..

[32] Malietzis G (2016). Low muscularity and myosteatosis is related to the host systemic inflammatory response in patients undergoing surgery for colorectal cancer. Ann. Surg..

[33] Prado CM (2008). Prevalence and clinical implications of sarcopenic obesity in patients with solid tumours of the respiratory and gastrointestinal tracts: a population-based study. Lancet Oncol..

[34] Villasenor A (2012). Prevalence and prognostic effect of sarcopenia in breast cancer survivors: The HEAL Study. J. Cancer Surviv..

[35] Shachar SS (2017). Body composition as a predictor of toxicity in patients receiving anthracycline and taxane-based chemotherapy for early-stage breast cancer. Clin. Cancer Res..

[36] Rossi F (2019). Evaluation of body Computed Tomography-determined sarcopenia in breast cancer patients and clinical outcomes: A systematic review. Cancer Treat Res. Commun..

[37] Prado CM (2009). Sarcopenia as a determinant of chemotherapy toxicity and time to tumor progression in metastatic breast cancer patients receiving capecitabine treatment. Clin. Cancer Res..

[38] Rier HN (2017). Low muscle attenuation is a prognostic factor for survival in metastatic breast cancer patients treated with first line palliative chemotherapy. Breast.

[39] Franzoi MA (2020). Computed tomography-based analyses of baseline body composition parameters and changes in breast cancer patients under treatment with CDK 4/6 inhibitors. Breast Cancer Res. Treat..

[40] van der Werf A (2018). Percentiles for skeletal muscle index, area and radiation attenuation based on computed tomography imaging in a healthy Caucasian population. Eur. J. Clin. Nutr..

[41] Caan BJ (2018). Association of muscle and adiposity measured by computed tomography with survival in patients with nonmetastatic breast cancer. JAMA Oncol..

[42] Goldhirsch A (2013). Personalizing the treatment of women with early breast cancer: Highlights of the St Gallen international expert consensus on the primary therapy of early breast cancer 2013. Ann. Oncol..

