# Supplementary material for: Computed tomography based analyses of body mass composition in HER2 positive metastatic breast cancer patients undergoing first line treatment with pertuzumab and trastuzumab
Source: Sci Rep. 2022 Mar 1;12:3385. doi: 10.1038/s41598-022-07143-1 (PMC8888586; doi:10.1038/s41598-022-07143-1)
Supplement: Supplementary file 2 — Supplementary Information 2. [file 41598_2022_7143_MOESM2_ESM.docx]

**Supplementary Table 1**. Univariate analysis for Overall Survival (OS) and all the parameters considered in the study

|  | **Median OS**  **months (95% CI)** | **long rank**  **p value** | **HR**  **(95% CI)** |
| --- | --- | --- | --- |
| **Overall** | 54.5 (35.1-nr) | - | - |
| **Age (years)** |  |  |  |
| <58 | 54.5 (29.3-nr) |  | Reference |
| ≥58 | nr | 0.900 | 0.93 (0.31-2.80) |
| **Stage** |  |  |  |
| I-III | 54.5 (28.5-nr) |  | Reference |
| IV | nr | 0.612 | 0.73 (0.22-2.44) |
| **Surgery** |  |  |  |
| No | nr |  | Reference |
| Yes | 54.5 (29.3-nr) | 0.775 | 0.84 (0.25-2.80) |
| **Histology** |  |  |  |
| Ductal | 54.5 (32.6-nr) |  | Reference |
| Other | nr | 0.418 | 0.43 (0.05-3.51) |
| **Metastatic site** |  |  |  |
| Other | 36.9 (29.3-nr) |  | Reference |
| Only Bone | nr | 0.058 | 0.17 (0.02-1.34) |
| **LDL (mg/dL)** |  |  |  |
| Normal (<100) | 54.5 (32.6-nr) |  | Reference |
| High (>100) | nr | 0.952 | 0.96 (0.28-3.32) |
| **Triglycerides (mg/dL)** |  |  |  |
| Normal (<150) | 54.5 (19.9-nr) |  | Reference |
| High (≥150) | nr | 0.432 | 0.62 (0.19-2.06) |
| **Menopausal status** |  |  |  |
| Pre | 35.1 (17.3-nr) |  | Reference |
| Post | nr | 0.230 | 0.49 (0.16-1.51) |
| **ER (%)** |  |  |  |
| Negative (0) | 29.3 (12.5-nr) |  | Reference |
| Positive (≥1) | nr | 0.094 | 0.32 (0.08-1.30) |
| **PgR (%)** |  |  |  |
| Low (≤20) | 35.1 (11.5-nr) |  | Reference |
| High (>20) | nr | 0.162 | 0.25 (0.03-2.04) |
| **Ki67 (%)** |  |  |  |
| Low (≤20) | nr |  | Reference |
| High (>20) | 35.1 (5.5-nr) | 0.137 | 4.63 (0.51-42.3) |
| **BMI (kg/m^2^)** |  |  |  |
| <25 | nr |  | Reference |
| ≥25 | 54.5 (28.5-nr) | 0.469 | 1.51 (0.49-4.62) |
| **SMI (cm2/m2)** |  |  |  |
| Normal (>40) | 54.5 (29.3-nr) |  | Reference |
| Sarcopenia (<40) | nr | 0.290 | 0.50 (0.14-1.84) |
| **SFI (cm2/m2)** |  |  |  |
| Normal (<82.97) | nr |  | Reference |
| High (>82.97) | 54.5 (19.9-nr) | 0.122 | 2.46 (0.76-7.99) |
| **VFI (cm2/m2)** |  |  |  |
| Normal (<37.1) | 54.5 (29.3-nr) |  | Reference |
| High (>37.1) | nr | 0.771 | 0.85 (0.28-2.55) |
| **TAFTI (cm2/m2)** |  |  |  |
| Normal (<118.82) | nr |  | Reference |
| High (>118.82) | 54.5 (28.5-nr) | 0.140 | 2.37 (0.73-7.70) |

**Supplementary Table 2.** Cox multivariate analysis of Overall Survival (OS)

|  | HR (95% CI) | p-value |
| --- | --- | --- |
| **TAFTI (cm2/m2)** |  |  |
| Normal (<118.82) | Reference |  |
| High (>118.82) | 1.85 (0.18-18.95) | 0.605 |
| **SFI (cm2/m2)** |  |  |
| Normal (<82.97) | Reference |  |
| High (>82.97) | 1.97 (0.26-14.87) | 0.509 |
| **BMI (kg/m^2^)** |  |  |
| <25 | Reference |  |
| ≥25 | 1.31 (0.24-7.12) | 0.753 |
| **ER (%)** |  |  |
| Negative (0) | Reference |  |
| Positive (≥1) | 0.32 (0.08-1.28) | 0.107 |
| **Menopausal status** |  |  |
| Pre | Reference |  |
| Post | 0.34 (0.10-1.13) | 0.079 |
